# Wound healing and longevity: Lessons from long-lived αMUPA mice

**DOI:** 10.18632/aging.100726

**Published:** 2015-02-24

**Authors:** Hagai Yanai, Dimitri Toren, Klemens Vierlinger, Manuela Hofner, Christa Nöhammer, Marco Chilosi, Arie Budovsky, Vadim E. Fraifeld

**Affiliations:** ^1^ The Shraga Segal Department of Microbiology, Immunology and Genetics, Ben-Gurion University of the Negev, Beer Sheva 84105, Israel; ^2^ AIT - Austrian Institute of Technology, ATU14703506, Vienna, Austria; ^3^ Department of Pathology, University of Verona, Verona, Italy

**Keywords:** wound healing, αMUPA mice, caloric restriction, aging, gene expression, growth-promoting pathways

## Abstract

Does the longevity phenotype offer an advantage in wound healing (WH)? In an attempt to answer this question, we explored skin wound healing in the long-lived transgenic αMUPA mice, a unique model of genetically extended life span. These mice spontaneously eat less, preserve their body mass, are more resistant to spontaneous and induced tumorigenesis and live longer, thus greatly mimicking the effects of caloric restriction (CR). We found that αMUPA mice showed a much slower age-related decline in the rate of WH than their wild-type counterparts (FVB/N). After full closure of the wound, gene expression in the skin of old αMUPA mice returned close to basal levels. In contrast, old FVB/N mice still exhibited significant upregulation of genes associated with growth-promoting pathways, apoptosis and cell-cell/cell-extra cellular matrix interaction, indicating an ongoing tissue remodeling or an inability to properly shut down the repair process. It appears that the CR-like longevity phenotype is associated with more balanced and efficient WH mechanisms in old age, which could ensure a long-term survival advantage.

## INTRODUCTION

The skin is the first and foremost natural barrier of the organism against foreign infection and hazards. As such, it is the most frequently injured tissue, and a quick repair of damaged skin is vital for the organism [1,2]. Though differing in specific mechanisms, the basic events during skin repair have much in common across a variety of wounded organs [3]. Not surprisingly, the skin is a widely used model for studying the basic and intricate process of wound healing (WH) [4–6].

In order to restore tissue integrity, several well-coordinated processes are mobilized, including the inflammatory responses, formation of granulation tissue, cell proliferation and final tissue remodeling [3,7]. Not less important is to shut down the wound healing mechanisms when the healing goal has been achieved. While skin wounds heal perfectly with no scars in early mammalian embryos [2], the regenerative capacity is drastically reduced after birth and continues to decline with advanced age [8–10]. Consequently, in adults, skin repair normally results in scar formation [2]. Deviations from regular skin WH (SWH) may lead to diverse pathological conditions, from slow or ineffective tissue repair (such as in cases of ischemic and diabetic ulcers) to fibroproliferative responses (such as hypertrophic scars).

Although there is no clear consensus on whether aging affects the quality of SWH, the rate of SWH is often used as one of the biomarkers for biological age and could be indicative of a longevity phenotype ([11] and references therein). However, a clear-cut answer as to whether the longevity phenotype is associated with accelerated SWH remains obscure. Even in case of calorie restriction (CR), one of the most successful longevity-promoting interventions in mammals (for recent reviews see [12,13]), the few studies conducted thus far did not bring about decisive results [14–16].

To address this issue, we investigated SWH in the long-lived transgenic αMUPA mice, a unique genetic model of extended lifespan. The αMUPA mice carry a transgene consisting of the full-length cDNA encoding the murine urokinase-type plasminogen activator (uPA) linked to the A-crystallin gene which is specifically expressed in the ocular lens [17]. Being initially generated in 1987 to investigate a potential role of uPA in eye pathologies (e.g. cataract and glaucoma), these transgenic mice were unexpectedly found to display a longevity phenotype. Compared to their wild type (WT) counterparts, the αMUPA mice *spontaneously* eat less when fed *ad libitum*, and live longer [18]. The αMUPA mice also maintain an overall young look and physical activity at advanced ages and show a significantly reduced rate of spontaneous and induced tumorigenesis [19,20]. Thus, the αMUPA mice share many common features with CR, yet are not hindered by several major drawbacks of CR such as hunger-induced stress and a need for individual housing (social stress). In view of using αMUPA mice as a CR-mimicking model to study the impact of CR on SWH, it is important to stress that the αMUPA mice strongly express the uPA in the ocular lens and ectopically in the brain but *not in the skin* [17], thus excluding the gene-specific effects on SWH.

## RESULTS

### 1. αMUPA mice preserve their body mass

**Figure 1:**
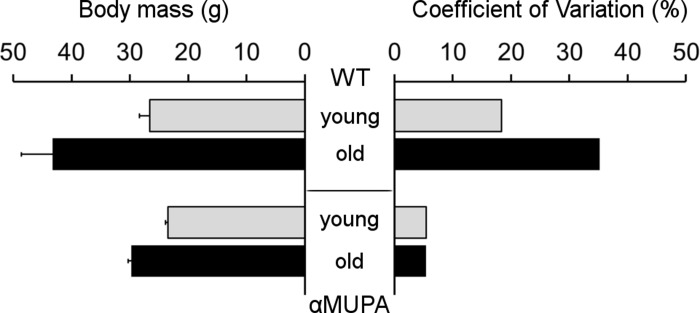
Body mass (left) and its variability (right) in αMUPA and WT mice of different age Body mass is presented as mean ± SEM. Variability was estimated by coefficient of variation (see Methods). N = 8 in each group.

As expected, αMUPA mice showed increased longevity compared to the WT. By 24 months, the survival of αMUPA was almost 95%, while only about 55% of WT mice from the initial colony survived. Moreover, the old αMUPA mice retained a youthful appearance and were physically active. They also preserved their body mass (BM), showing only a slight increase in average BM as compared to the young αMUPA animals (29.8 ± 0.6 g vs. 23.5 ± 0.5 g; *p* = 7.7E−07) and no significant difference with young WT mice (p = 0.114) (Fig. 1).

In contrast, the average BM of old WT mice was almost twofold higher than that of the young animals (43.2 ± 5.4 g vs. 26.6 ± 1.7 g; *p* = 0.017). Remarkably, both young and old αMUPA mice displayed a high homogeneity of BM, reflected by a very low coefficient of variation (CV; 5.4% and 5.5%, respectively), while WT mice displayed a much higher variability which markedly increased with age (from 18.4% in the young to 35.2% for the old) (Fig. 1).

### 2. Aged αMUPA mice preserve the rate of wound healing

**Figure 2:**
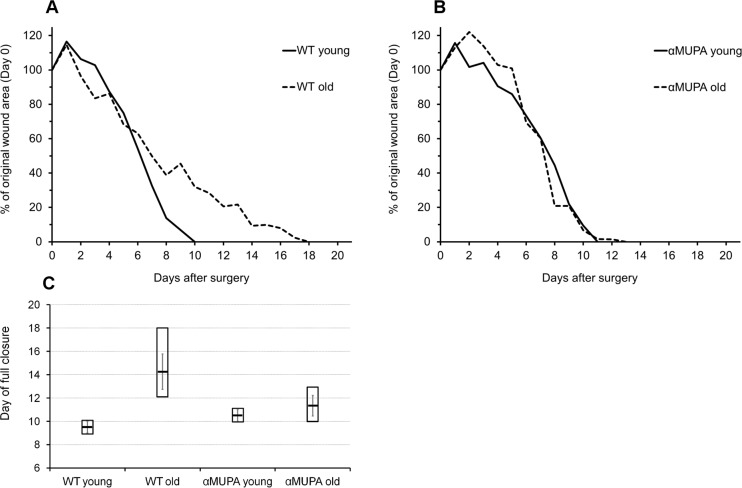
Time-course of wound closure in αMUPA and WT mice of different ages Measurements were made on a daily basis, from Day 0 to Day 21 after surgery. (**A**) Wild type (**B**) αMUPA. (**C**) Day of full closure presented as mean (central line) ± SEM (whiskers) and Min/Max (box).

In order to evaluate the impact of aging and the longevity phenotype on regular SWH, a round full-thickness wound was administered to young (3-4 mo) and old (24 mo) αMUPA mice and their age-matched parental WT counterparts. In young mice of both strains, full closure of head excisional wounds occurred by Day 8-11 after surgery (9.5 ± 0.7 and 10.5 ± 0.7 for WT and αMUPA, respectively; *p* > 0.6) (Fig. 2). Similar values (10-13 days, 11.3 ± 1.5) were observed in the old αMUPA mice (*p* > 0.3; old αMUPA vs. young αMUPA) (Fig. 2). In contrast, the period for SWH in old WT mice was significantly longer and more variable, reaching 12-18 days (15.3 ± 2.6, *p* < 0.05; old WT vs. young WT) (Fig. 2). As in the case of BM, the variation in wound closure rate increased in the old WT mice, but remained more uniform in the old αMUPA (Fig. 2).

### 3. Histological assessment of the skin during wound healing

**Figure 3:**
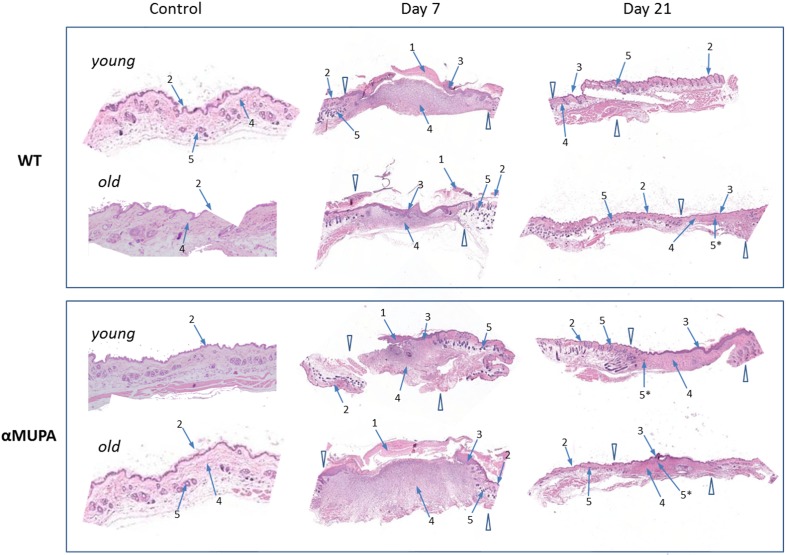
Histological examination of skin wound healing in αMUPA and WT mice Hematoxylin and eosin [H&E] staining of the skin samples of Day 0 (control), Day 7 (early re-epithelialization) and Day 21 (full wound closure) after operation. Magnification: 4x. Arrowheads indicate the wound edges. Arrows: 1 – granulation tissues; 2 – normal epidermis; 3 – hyperplastic epidermis; 4 – fibroblasts; 5 – hair follicles; 5* – new hair follicle formation within re-epithelialized edges.

Histological assessment of the skin samples showed that despite of the slower WH in old WT mice (Fig. 2), there were no overt morphological differences between the age or strain groups with regard to the formation of granulation tissue and early re-epithelialization (Day 7). Also, by Day 21, all operated mice displayed full wound closure, with no obvious differences in the scar and surrounding tissues (Fig. 3). Thus, independently of age or genetic background, all animals reached a similar end result – wound closure with formation of a scar tissue.

### 4. Primary dermal fibroblasts from aged αMUPA mice display higher motility

**Figure 4:**
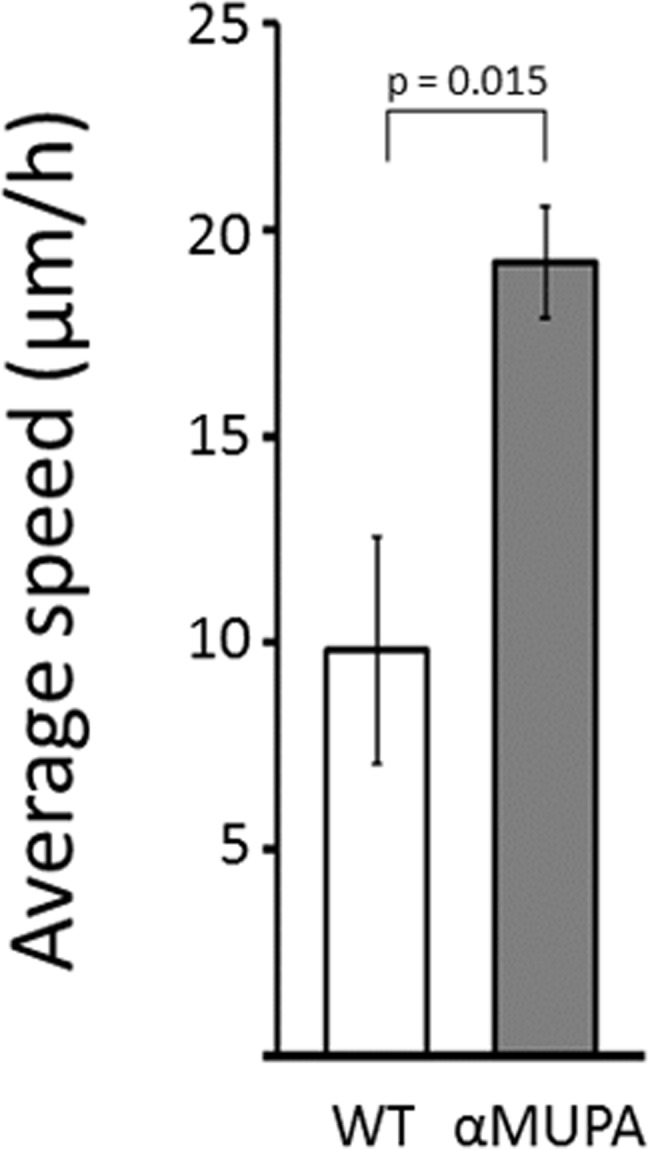
Average speed of fibroblasts derived from aged αMUPA and WT mice that are involved in in vitro gap closure Calculation of speed was based on a scratch assay and measured with a brightfield microscope at x4 and x10 magnification. Distance was calculated using the ImageJ software.

The rate of SWH is greatly attributed to the potential of dermal fibroblasts to migrate into the injured area, which can be assessed *in vitro* by measuring cell motility. As the strain-dependent difference in SWH rate was evident only in aged mice, we tested the motility of primary cultures of dermal fibroblasts derived from this age group, using the scratch assay. This *in vitro* WH model is a simple and straight forward method to study cell motility as the artificial gap created is mostly closed by fibroblast migration rather than cell proliferation [21]. As seen in Fig. 4, the average speed of dermal fibroblasts participating in closing the *in vitro* gap (created by the scratch) was approximately twofold higher in aged αMUPA vs. aged WT (p = 0.015). Of note, cells derived from old αMUPA mice showed a much lower variability in the average speed than cells from old WT mice (reflected by a CV of 0.156 and 0.624, respectively).

### 5. Age- and strain-related differences in gene expression

**Figure 5:**
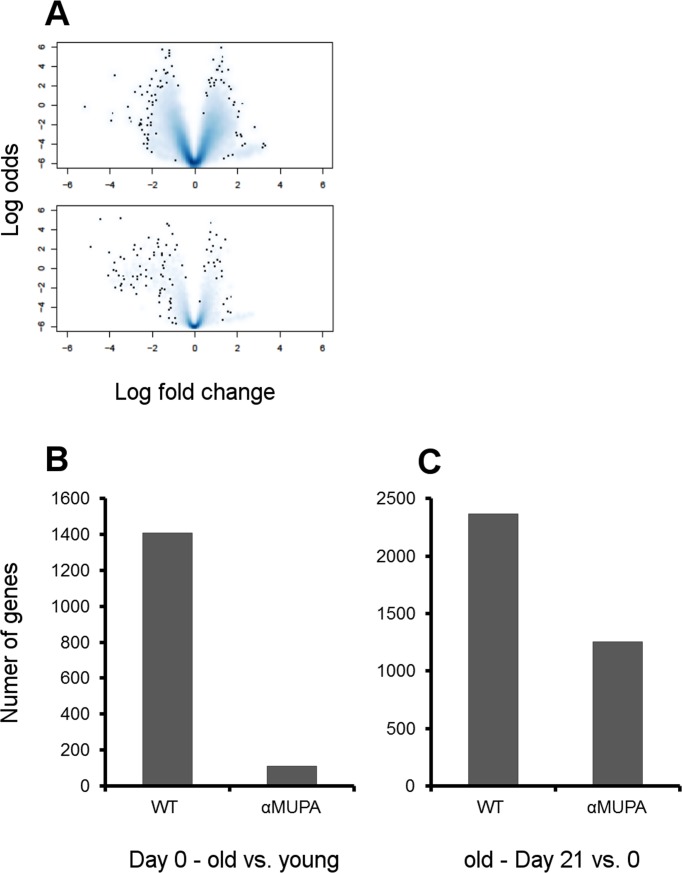
Age- and strain-related differences in gene expression in intact and healed skin (**A**) Volcano plots (effect size) of aged groups vs. young groups. Effect size was plotted as log2 of fold change vs. significance (B-value, i.e. log odds ratio of a gene being differentially expressed). Single dots represent individual genes and clouds represent multiple genes where cloud density correlates to the number of genes on the same pixel. WT – upper panel; αMUPA – lower panel. (**B**) The number of genes differentially expressed in aged vs. young animals at Day 0 (intact skin) (p < 0.05). (**C**) The number of genes differentially expressed in the skin of aged animals after histologically confirmed full closure of the wound (Day 21 vs. Day 0; p < 0.05).

When comparing gene expression in the intact skin, we surprisingly found that there were no significant differences between young αMUPA and WT mice (0 genes differentially expressed with at least twofold changes and *p*-value < 0.01; even under less stringent conditions [*p*-value < 0.05] there were only 2 differentially expressed genes). Alongside with the histological results (see above), this indicates that the skin of young αMUPA mice is fundamentally the same as that of their WT counterparts. However, the age-related changes in gene expression were much more pronounced in WT vs. αMUPA mice (Fig. 5A,B).

In WT, the gene expression profile of aged vs. young mice was enriched in several pathways involved in basic cell processes (such as DNA maintenance, cell cycle, apoptosis, ubiquitin-mediated proteolysis pathways) and immune functions (Suppl. Table 1). In contrast, aged αMUPA mice display a much smaller number of differentially expressed genes as compared to the young (Fig. 5A,B), with a far lower number of enriched pathways (Suppl. Table 1). The only common pathway that was found to be enriched in aged αMUPA and WT was the tight junction pathway.

### 6. Aged αMUPA mice display more balanced molecular mechanisms of wound healing

In contrast to αMUPA mice, the WT mice exhibited significant age-related differences in gene expression, both in intact animals and in the course of SWH (Fig. 5, Suppl. Tables 1, 2). The enrichment analysis revealed that in aged WT mice, in spite of the histologically confirmed full wound closure (Day 21), numerous pathways are still significantly activated as compared to the intact skin (Table 1, Suppl. Table 2). This was especially noted for growth-promoting pathways including the Insulin, mTOR, ErbB, Wnt, MAPK, and VEGF signaling pathways, and various cancer-associated pathways. Along with growth-promoting pathways, the apoptosis and cell-cell/ECM interaction (focal adhesion and adherens junction) pathways were also enriched in the differentially expressed genes. Altogether, activation of these pathways indicates ongoing tissue remodeling. Unlike old WT mice, the old αMUPA mice did not display any significant enrichment in growth-promoting pathways after wound closure (Suppl. Table 2), indicating more balanced WH mechanisms.

**Table 1:**
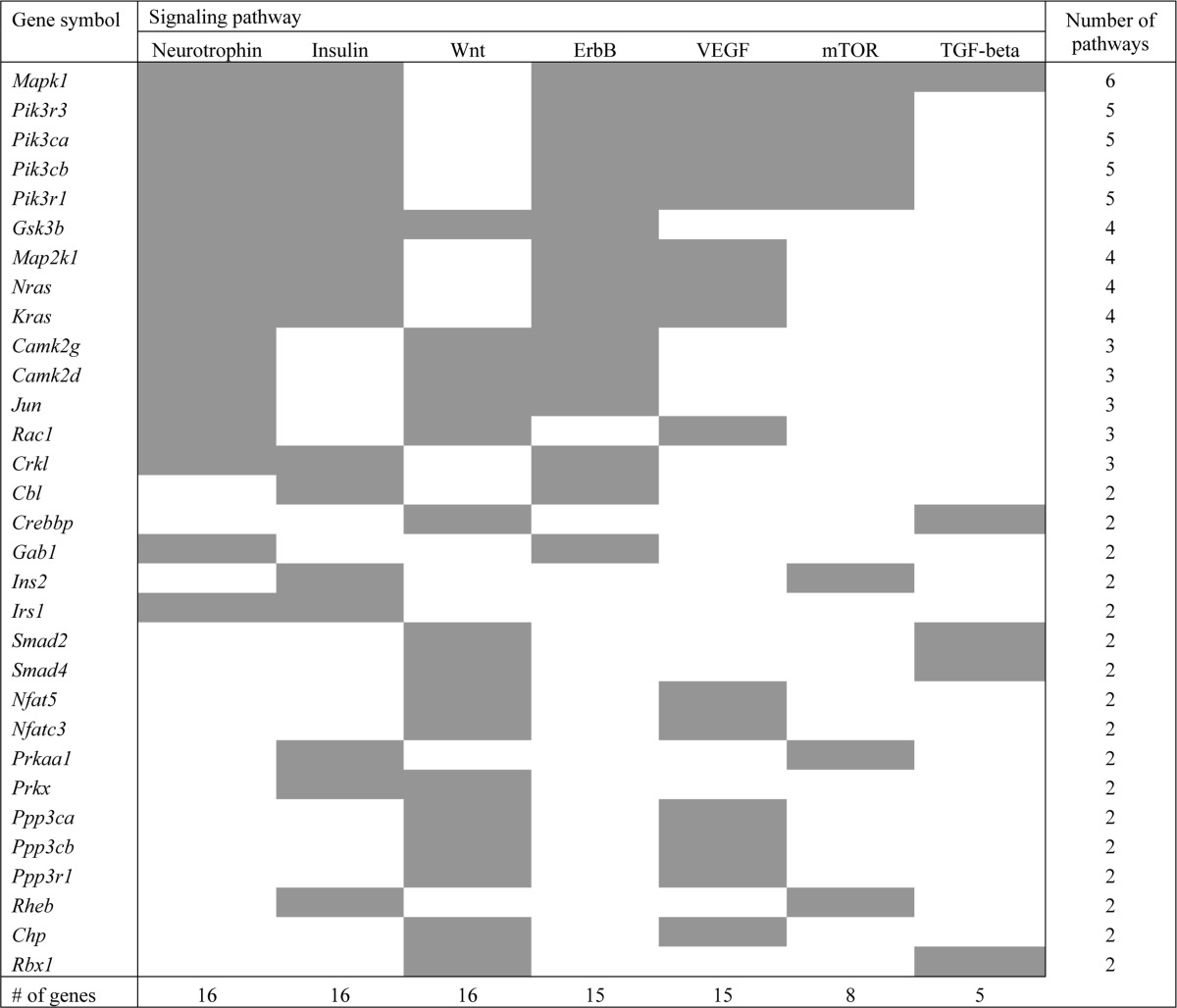
Growth-promoting pathways related genes upregulated at Day 21 vs. Day 0 in old WT mice

No significant KEGG pathway enrichment was observed in young mice after full wound closure (Day 21).

In Table 1 are presented KEGG growth-promoting pathways that were found to be enriched among differentially expressed genes between Day 21 and Day 0 in old WT mice (with adjusted *p*-value < 0.05). Only genes which are common to at least two pathways are listed. For a full list of enriched pathways and gene IDs, see Suppl. Tables 1, 2, 3.

**Figure 6:**
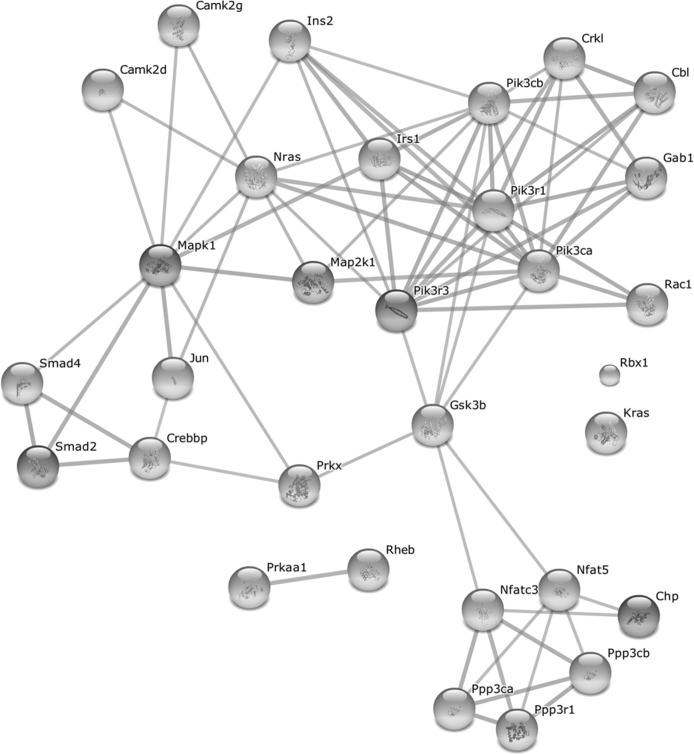
Protein-protein interaction network formed by upregulated genes common to at least two growth-promoting pathways after full wound closure in old WT mice For gene list and associated pathways see Table 1. Network was created with STRING 9.1 ([22], http://string-db.org/) using high confidence score settings based on experimental data and databases. The interactivity of the 31 genes from Table 1 exhibits an extraordinarily significance (p < 10^−25^).

As the above pathways share common genes which might ensure a cross-talk between the pathways, we further analyzed the differentially expressed genes which are common to at least two pathways (mentioned in Table 1). As shown in Fig. 6, these common proteins form a highly interconnected protein-protein interaction network among themselves, thus highlighting an orchestrated activation of the growth-promoting pathways in old WT mice. Indeed, all but one of these genes are upregulated in old WT mice after full wound closure (Suppl. Table 3). Several key proteins in this network are also involved in other enriched pathways observed at Day 21. This includes phosphatidylinositol 3-kinases involved in apoptosis and focal adhesion pathways; protein phosphatases for apoptosis pathway; MAP kinases for focal adhesion and adherens junction pathways; and transcription factors CREB binding protein and Smad 2 and 4 for adherens junction pathway. Interestingly, these genes also tend to be upregulated in the intact skin of old WT mice (20 of 31 with p < 0.05), indicating an age-related predisposition of the aging skin for growth promotion. In contrast, in old αMUPA mice, only five of these 31 genes (*Cbl*, *Jun*, *Pik3r3*, *Ppp3r1*, and *Camk2d*) are upregulated after full wound closure and none is differentially expressed in the intact skin (Suppl. Table 3).

## DISCUSSION

The optimal outcome of the WH response is the complete regeneration of tissue structure and function, as occurs in several species from diverse taxa (e.g., salamander, axolotl, hydra, planaria) and in early mammalian embryos [23]. Yet, in postnatal mammals, tissue repair is usually achieved by a combination of an inflammatory response with rapid scarring repair, instead of the full albeit slower tissue regeneration [3]. Such a trade-off, favoring speed of restoration over functionality, is probably imperative in a variety of mammalian tissues, especially for the skin in the wild, where a quick repair of damaged skin is vital for the protection of the organism from pathogen invasion [2]. Thus, it is reasonable to assume that accelerated skin repair could be positively associated with a longevity phenotype. In order to examine this hypothesis, we utilized the transgenic αMUPA mice, a unique mammalian model of extended lifespan that mimics the longevity-promoting effects of CR, but without the well-known downsides such as hunger or individual housing stress [17].

It should also be noted that out of several *in vivo* wound healing techniques in use, the back models are the most popular. This is also the case in aging studies in rodents ([11] and references therein). However, wound closure in the rodent back models relies heavily on tissue contraction and to a lesser degree on the formation of new tissue [3]. This mostly occurs because the rodent skin has a subcutaneous thin muscle layer (*panniculus carnosus*) which is almost completely lacking in humans, except for the platysma in the neck [6]. Our model, as a result of the splinting effect of the skull, depends mostly on the formation of granulation tissue and re-epithelization [3]. Such a situation is more common for humans [6], and thus, the murine head punch model utilized in our study is more relevant for human studies.

Three major findings of this study are discussed below.

***“Slow but steady wins the race”***. Our functional (quantitative follow-up of wound closure) analysis revealed that in the young age, the αMUPA mice were not superior over their parental WT strain with regard to their skin WH capacity. However, the αMUPA showed a much slower age-related decline in the rate of SWH compared to the WT, so that, in aged mice, the differences between αMUPA and WT become evident (Fig. 2). Although αMUPA mice share many features with CR mice [17], previous studies in CR models of SWH rate were not fully consistent with our results. In these studies, reduced calorie intake did not grant any advantages for the rate of SWH in young animals (mice [14], rats [15,16] and monkeys [15]), which is in line with our results. However, only a slight (but not significant) increase in the healing rate was observed in middle-aged (18-month-old) CR Wistar rats in comparison to *ad libitum* fed animals [15]. In the same study, a clear trend toward faster closure was observed for old CR-fed rhesus monkeys. Of note, in all these studies, SWH was tested in the dorsal area which, as described above, relies largely on contractile repair rather than on the formation of granulation tissue and re-epithelization (as in our study). It seems therefore that the back SWH model might to some extent mask the beneficial effects of CR on SWH in aged animals. Yet, these effects are clearly evident in the head punch model. One plausible explanation is that low calorie consumption primarily attenuates the age-related decrease in scar tissue formation, and to a lesser degree affects the initial stages of SWH, i.e. wound contraction.

Our results indicate that the relationship between the rate of SWH and the longevity phenotype is of an age-dependent nature and manifests only later in life. This conclusion is also strengthened by our recent analysis of SWH studies conducted on various murine genetic models of altered lifespan, i.e., premature aging or longevity phenotype [11]. We have shown a positive relation between the rate of SWH and the longevity of mice, yet this trend was true only for advanced but not the young ages. As in CR studies, the rate of SWH in the young animals was not indicative of the “longevity potential” [11]. Thus, the ability to preserve the rate of SWH up to an old age, but not necessarily a high WH rate in the young, appears to be associated with a longevity phenotype.

***“All roads lead to Rome”***. Despite the slower SWH in aged WT mice and the variations in gene expression between groups, all animals reached a similar end result – full closure of wounded skin, formation of scar tissue without development of hypertrophic, fibroproliferative conditions. No gross morphological differences between the age or strain groups were observed. In a broader sense, our findings exemplify that such a complicated process as wound healing, possesses remarkable plasticity which may ultimately ensure the final goal – recovery of tissue integrity.

***"Begin at the beginning and go on till you come to the end; then stop” (Lewis Carroll)***. The injury-induced activation of growth-promoting and cell-cell/ECM interaction pathways in aged WT mice was evident even after a histologically confirmed full closure of the wound (Day 21), indicating an *ongoing* tissue remodeling. As such, this inability to properly shut down the wound healing process in the old, or in other words, insufficient response to feedback signals, seems to be quite typical for many age-related conditions and perhaps for aging as a whole. For example, this sort of dysregulation has been reported for chronic inflammation [24], continuation of the developmental program due to signal resistance in hypothalamic feedback [25–28], and more. Continuous over-healing has also been proposed as one of the underlying mechanisms in age-related pathologies including atherosclerosis [29,30], pulmonary fibrosis [31], and cancer [32]. On the other hand, a good example of a well-balanced feedback is the phenomenon of “early contact inhibition” in naked mole-rats known for their exceptional longevity and resistance to cancer [33]. Our results suggest that the longevity phenotype (eg, αMUPA mice) appears to be associated with a more balanced WH response and more reliable feedback regulation in general.

Supporting this notion is the remarkable homogenousity of αMUPA mice. In our study, this was illustrated by the low variability of body mass (Fig. 1), the rate of skin wound healing (Fig. 2), and the speed of *in vitro* fibroblast migration (Fig. 4). This was quantified by a much lower, scaleless coefficient of variation (CV) of the above parameters for αMUPA vs. WT mice. Remarkably, the differences in CV values were particularly noted in old animals which generally display a higher variability. This observation could have far reaching consequences. According to the Reliability Theory of Aging and Longevity [34], aging is a result of a gradual decrease in the reliability of different systems in the organism. The CV has long been used in engineering for evaluation of the reliability of technical systems: the lower the CV, the higher the reliability. From this point of view, the increased longevity of αMUPA mice and preserved rate of wound healing could be explained, at least in part, by a higher reliability of regulatory mechanisms including the system of skin repair.

In essence, it is attractive to suggest that counteracting the age-related decline in feedback responses could be one of the keystones of longevity-promoting interventions, CR included.

## MATERIALS AND METHODS

### Animals

Transgenic homozygous αMUPA mice and their parental WT counterparts (the NIH inbred mouse strain FVB/N) were kindly provided by Prof. Ruth Miskin. Animals were propagated and maintained at the Transgenic Mouse Facilities of the Weizmann Institute of Science according to the NIH guide for care and use of laboratory animals. The experiments were carried out on female αMUPA and WT mice of two age groups: young (4–5 months) and old adults (24–25 months) (Table 2). Only animals with no overt pathological manifestations as confirmed by autopsy examination were included in the experiments. To minimize the influence of circadian variation, the surgery, biopsy collection and digital photography of the wounds were performed during the light hours between 10:00-12:00. Regular SWH was analyzed in 32 mice subjected to a full-thickness surgical head excision (see next section). All experiments were approved by the Israel Ministry of Health Committee for Animal Experimentations (Authorization number: IL-82-12-2009).

**Table 2:** Distribution of mice and collected skin samples by murine strain, age, and experimental group (time point of harvesting the skin biopsies after surgery)

Experimental groups	αMUPA		FVB/N (WT)	
	Young adult	Aged	Young adult	Aged
Day 0	Self-control [*]			
Day 7	4 [4*]	4 [4*]	4 [4*]	4 [4*]
Day 21	4 [4*]	4 [4*]	4 [4*]	4 [4*]
Number of mice	8	8	8	8
Number of skin samples	16	16	16	16

[*] samples taken at the time of surgery

### Head excision model of skin wound healing

Mice were intra-peritoneally anesthetized before surgery with xylacine 5 mg/kg and ketamine 100 mg/kg of body mass. Full-thickness wounds were generated on the crown of the skull [3] using an 8-mm trephine (Punch Biopsy). The injured tissue was then excised down to the bone with curved sharp scissors. The excised skin served as self-control and was immediately divided into three pieces for histological analysis, transcriptomics, and backup. Specimens designated for transcriptomics analysis and those serving as backup were snap-frozen in liquid nitrogen and stored at −80 °C. Samples designated for histology analysis were placed in paraformaldehyde 4% solution and later treated as described in the next section. The posterior end of the non-wounded area was marked for histological sections with a 5/0 Prolene filament. A semi-occlusive dressing (TegadermTM, 3MTM, health care) was applied to the wound after skin excision to avoid desiccation. The dressing was attached to the skin at the wound margins via two 5-0 fibrillous sutures. The transparent nature of the material allowed visual examination and photography of the wound. The wounds were left to heal by secondary intention, i.e., the wound edges were not closed with sutures. On Day 7 (late stage of granulation tissue formation and early re-epithelialization) and Day 21 (full wound closure and scar formation), the mice of corresponding groups were anesthetized and skin samples of the wound area were excised and processed as described above. Mice were then sacrificed using a lethal dose of the above anesthetics and processed for autopsy examination.

### Histological examination of skin samples

After surgery (Day 0) and at indicated time points (Day 7 and Day 21), the head skin biopsies were collected as described above and the posterior half from each sample was placed in 4% paraformaldehyde overnight and then stored in 70% ethanol at 4 °C, pending paraffin fixation, 3-μm sectioning and Hematoxylin-eosin (H&E) staining. Pictures were taken at x2 and x20 magnifications. All the histologic examinations were performed as a double-blind study.

### Measurement of wound area

To follow up the wound closure, pictures of the wound area were taken every day after surgery, at the same hour of the day in 24-h intervals. Briefly, the mice were placed in a well lit room, on a table with a white background. At least 5 digital photographs (Cannon IXY, 4 M) of the wound area were taken for each mouse at indicated time points. Two types of pictures were taken: (1) Close-up pictures for thorough visual evaluation of the wound/scar; (2) pictures taken from a distance of 25 cm while a ruler was aligned next to the wound (for wound area measurement). To minimize any possible biases, the morphometric analysis of wound closure was performed as a double-blind study. Quantification of the wound area was carried out using the open source NIH ImageJ v1.43 software. Scale was set separately for each image, according to the ruler, using the ImageJ straight tool.

### *In vitro* scratch assay

Primary cultures of dermal fibroblasts were derived from the mouse skin as described elsewhere [35]. Cells were cultivated in Dulbecco's Modified Eagle Medium (DMEM) supplemented with 10% Fetal Bovine Serum (FBS), 0.1 mg/ml streptomycin and 0.29 mg/ml Glutamate (Biological Industries Ltd.). Scratch assay was performed on confluent cell cultures of early passages as described in Liang et al [21]. Pictures were taken under an inverted microscope (at x4 and x10) and quantified using the ImageJ software [36]. Average speed of fibroblast migration was calculated as distance covered by cells per hour.

### Gene expression array

Approximately 30 mg of tissue was lysed in RLT-buffer (Qiagen p/n 80204) in a FastPrep FP120 instrument (Qbiogene p/n 6001-120) and extracted using Qiagen All-Prep DNA/RNA extraction kit (Qiagen p/n 80204) according to the manufacturer's instructions. RNA quality was assessed by the Agilent 2100 Bioanalyser (p/n G2938C). RNA samples were labeled using the Agilent low RNA input fluorescent linear amplification Kit (p/n 5184-3523) according to the manufacturer's instructions. Briefly, 200 ng of total RNA was reverse transcribed. Amplification and labeling were performed by T7-polymerase *in vitro* transcription to produce Cy3-labeled cRNA. The dye incorporation rate was assessed with a Nanodrop ND-1000 spectrophotometer and was consistently >9 pmolCy3/μgRNA. Single color hybridizations were carried out using the Agilent Gene Expression Hybridization Kit (p/n 5188-5242 according to the manufacturer's instructions. 1650 ng of cRNA was subjected to fragmentation (30 min at 60 °C) and then hybridized to 4x44K Human Whole-Genome 60-mer oligo-chips (G4112F, Agilent Technologies) in a rotary oven (10 rpm, 65C, 17 h). Slides were disassembled, washed in solutions I and II, according to the manufacturer's instructions, and dried using Acetonitril. Scanning was done by an Agilent microarray scanner (p/n G2565BA) followed by Agilent Feature Extraction Software. Raw data was background corrected, quantile normalized and log2-transformed using R/Bioconductor (http://www.bioconductor.org; Gentleman et al, 2004). Differential expression was calculated using a linear model of the limma software package [37].

### Pathway enrichment and protein-protein interaction network

The k-means clustering algorithm was employed to group concordantly expressed genes (utilizing a linear model on the basis of F-statistic; *p* < E-4). Each of these gene clusters was subsequently subjected to Gene Set Enrichment Analysis against the KEGG pathway database with a cutoff value of p < 0.05. Subsequently, selected gene lists were analyzed for protein protein interaction networks using STRING 9.1 ([22], http://string-db.org/), only significantly differentially expressed genes with adj. P < 0.05 and at least two-fold change in expression were used. Network created based solely on experimental evidence and databases, constructed on high confidence score settings (0.7) and presented in confidence view.

## SUPPLEMENTARY TABLES






