# Erratum: Wound healing and longevity: Lessons from long-lived αMUPA mice

**DOI:** 10.18632/aging.100916

**Published:** 2016-02-28

**Authors:** Hagai Yanai, Dimitri Toren, Klemens Vierlinger, Manuela Hofner, Christa Nöhammer, Marco Chilosi, Arie Budovsky, Vadim E. Fraifeld

**Aging (Albany NY) 2015; 7(3)**: **167-176**.

PMCID: PMC4394728 PMID: 25960543

In this Article, the additional affiliation is added for Arie Budovsky, a co-author of this manuscript:

http://www.impactaging.com/papers/v7/n3/pdf/100726.pdf. The additional affiliation 4 is provided here:

Hagai Yanai^1^, Dimitri Toren^1^, Klemens Vierlinger^2^, Manuela Hofner^2^, Christa Nohammer^2^, Marco Chilosi^3^, Arie Budovsky^1,4^, and Vadim E. Fraifeld^1^

^1^The Shraga Segal Department of Microbiology, Immunology and Genetics, Ben-Gurion University of the Negev, Beer Sheva 84105, Israel

^2^AIT - Austrian Institute of Technology, ATU14703506, Vienna, Austria

^3^Department of Pathology, University of Verona, Verona, Italy

^4^**Judea Regional Research and Development Center, Carmel, Israel**

